# Relationship between Energy Balance and Circulating Levels of Hepcidin and Ferritin in the Fasted and Postprandial States

**DOI:** 10.3390/nu13103557

**Published:** 2021-10-11

**Authors:** Wandia Kimita, Sakina H. Bharmal, Juyeon Ko, Jaelim Cho, Maxim S. Petrov

**Affiliations:** School of Medicine, University of Auckland, Auckland 1023, New Zealand; wandia.kimita@auckland.ac.nz (W.K.); s.bharmal@auckland.ac.nz (S.H.B.); ju.ko@auckland.ac.nz (J.K.); j.cho@auckland.ac.nz (J.C.)

**Keywords:** iron metabolism, hepcidin, ferritin, ghrelin, leptin, new-onset diabetes, pancreatitis

## Abstract

Markers of iron metabolism are altered in new-onset diabetes, but their relationship with metabolic signals involved in the maintenance of energy balance is poorly understood. The primary aim was to explore the associations between markers of iron metabolism (hepcidin and ferritin) and markers of energy balance (leptin, ghrelin, and the leptin/ghrelin ratio) in both the fasted and postprandial states. These associations were also studied in the sub-groups stratified by diabetes status. This was a cross-sectional study of individuals without disorders of iron metabolism who were investigated after an overnight fast and, in addition, some of these individuals underwent a mixed meal test to determine postprandial responses of metabolic signals. The associations between hepcidin, ferritin, and leptin, ghrelin, leptin/ghrelin ratio were studied using several multiple linear regression models. A total of 76 individuals in the fasted state and 34 individuals in the postprandial state were included. In the overall cohort, hepcidin was significantly inversely associated with leptin (in the most adjusted model, the β coefficient ± SE was −883.45 ± 400.94; *p* = 0.031) and the leptin/ghrelin ratio (in the most adjusted model, the β coefficient ± SE was −148.26 ± 61.20; *p* = 0.018) in the fasted state. The same associations were not statistically significant in the postprandial state. In individuals with new-onset prediabetes or diabetes (but not in those with normoglycaemia or longstanding prediabetes or diabetes), hepcidin was significantly inversely associated with leptin (in the most adjusted model, the β coefficient ± SE was −806.09 ± 395.44; *p* = 0.050) and the leptin/ghrelin ratio (in the most adjusted model, the β coefficient ± SE was −129.40 ± 59.14; *p* = 0.037). Leptin appears to be a mediator in the link between iron metabolism and new-onset diabetes mellitus. These findings add to the growing understanding of mechanisms underlying the derangements of glucose metabolism.

## 1. Introduction

The central nervous system integrates metabolic signals from peripheral tissues to maintain energy balance. Leptin and ghrelin are key metabolic signals in this regard. Leptin is a hormone secreted primarily from the adipose tissue to relay the status of the energy stores to the central nervous system [1,2]. Leptin acts by stimulating the anorexigenic pathways in the appetite-regulating nucleus of the human hypothalamus, transmitting this information as peripheral signals that cause appetite suppression, limit food intake/energy consumption, and regulate energy expenditure [3,4]. Ghrelin is a hormone secreted mainly in the fundus of the stomach in adults (though the main source of ghrelin expression during perinatal life is the pancreas [5,6]) in response to a negative energy balance such as during fasting and starvation. Ghrelin primarily stimulates orexigenic pathways that trigger the feeling of hunger and increase appetite by affecting the same hypothalamic arcuate neurons engaged by leptin [7,8,9]. Ghrelin and leptin are antagonistic signals, and the leptin/ghrelin ratio is deemed a biomarker of metabolic adaptation to energy balance [10,11].

Iron is an essential inorganic micronutrient that plays a central role in numerous metabolic processes [12]. At the cellular level, iron acts as an indispensable co-factor to enzymes that catalyse metabolic pathways [13]. Iron levels are tightly regulated owing to their reactive nature and the potential to produce highly reactive free radicals that cause damage to cellular components [14]. Therefore, dysregulation of iron homeostasis plays an important role in the pathogenesis of metabolic diseases (including impaired glucose metabolism) [14]. Specifically, moderate to high body iron stores (as evidenced by increased serum ferritin levels) are a risk factor for type 2 diabetes, non-alcoholic fatty liver disease, and metabolic syndrome [15,16,17]. Likewise, deranged levels of circulating hepcidin (the liver-derived key regulator of iron metabolism) have been found in metabolic disorders [18,19,20,21,22]. Metabolic disorders are also often characterised by altered energy balance, and restoration of energy balance is emerging as a cornerstone of diabetes remission [23,24].

Several animal studies have reported a strong relationship between iron metabolism and serum leptin and ghrelin [25,26]. Dietary iron supplementation has been found to increase appetite and deplete leptin levels in rodents [26]. Further, increased iron stores (i.e., serum ferritin) were demonstrated to down-regulate leptin expression directly [26,27], and ghrelin was found to be involved in the regulation of the expression of ferroportin 1 (the transmembrane protein that allows entry and exit of iron into cells) [28]. Human studies have mainly focused on people with disorders of iron metabolism. For instance, thalassemia major patients with iron overload (as a result of multiple blood transfusions) were often reported to have markedly lower leptin levels when compared with healthy controls [29,30,31]. On the other hand, iron deficient people (who often have an accompanying poor appetite) were shown to have lower levels of ghrelin and hepcidin when compared with healthy controls [32], and dietary iron supplementation improved their appetite scores [33].

Despite the possible role of iron metabolism in the regulation of leptin and ghrelin, little is known about this relationship in humans with deranged glucose metabolism (who have no overt disorders of iron metabolism), possibly owing to the methodological difficulties of studying these populations. New-onset prediabetes/diabetes has been shown to occur in around 2 out of 5 individuals after an attack of acute pancreatitis (the most common disease of the exocrine pancreas) in the 2020 prospective longitudinal cohort study called LACERTA [34]. In comparison with other common types of diabetes (such as type 2 diabetes and type 1 diabetes), the cause of glucose abnormalities after acute pancreatitis is known (i.e., an attack of acute pancreatitis), and this presents an appealing opportunity to study a relatively homogenous population suitable for investigating the cross-talk between energy balance and iron metabolism in humans with no disorders of iron metabolism [35,36,37,38,39].

The primary aim of the present study was to explore the associations between markers of iron metabolism (specifically, hepcidin and ferritin) and metabolic signals involved in the maintenance of energy balance (specifically, leptin, ghrelin, and the leptin/ghrelin ratio) in individuals after an attack of acute pancreatitis, in both the fasted and postprandial states. The secondary aim was to investigate these associations in the sub-groups stratified by habitual dietary intake of iron and diabetes status.

## 2. Methods

### 2.1. Study Design and Participants

This cross-sectional study, which was part of the ANDROMEDA project, included adults (aged at least 18 years) with a history of acute pancreatitis (defined based on the international guidelines [40]). Participants were excluded if they had a history of iron overload, iron deficiency, haemochromatosis, chronic pancreatitis, post-endoscopic retrograde cholangiopancreatology pancreatitis, intraoperative diagnosis of pancreatitis, pregnancy or lactating at the time of pancreatitis or after, malignancy, or a recurrent attack of acute pancreatitis within three months of enrolment in the study. The study protocol was approved by the Health and Disability Ethics Committee (13/STH/182). All participants provided written informed consent.

### 2.2. Assessment of Dietary Iron Intake

The comprehensive EPIC-Norfolk food frequency questionnaire was used to record the participants’ usual food intake in the preceding 12 months [41,42]. Total dietary iron intake (mg/day) and total energy intake (kcal/day) were estimated using the standardised EPIC Nutrient Database [43]. Measurement of supplemental iron intake was beyond the scope of the data collection tool and, therefore, not considered in the present study. Iron intake was categorised into “insufficient” or “sufficient” iron intake as per sex- and age-specific recommended dietary allowances (RDAs) laid down by the Institute of Medicine (USA) [44]. The iron intake RDA for men and postmenopausal women was 8 mg/day, whereas that for premenopausal women was 18 mg/day. Participants with iron intake less than the RDA were deemed to have “insufficient” iron intake in the present study, whereas those with iron intake greater than or equal to the RDA were deemed to have “sufficient” iron intake.

### 2.3. Ascertainment of Diabetes Status

Diabetes status was categorised as normoglycaemia, type 2 prediabetes/diabetes mellitus (T2DM), and new onset prediabetes/diabetes after acute pancreatitis (NODAP) and defined according to the “DEP criteria” [45]. Normoglycaemia was defined as fasting blood glucose (FBG) <5.6 mmol/L (<100 mg/dL) and/or haemoglobin A1c (HbA1c) <5.7% (<39 mmol/mol). The T2DM group included individuals with HbA1c ≥5.7% (≥39 mmol/mol) before, during hospitalisation for acute pancreatitis, and within three months afterwards. The NODAP group included individuals with FBG ≥5.6 mmol/L (≥100 mg/dL) and/or HbA1c ≥5.9% (≥39 mmol/mol). FBG ≥5.6 mmol/L (≥100 mg/dL) during hospitalisation was not taken into account for the purpose of the above categorisation due to the possibility of stress-induced hyperglycaemia during an attack of acute pancreatitis [46].

### 2.4. Mixed Meal Test

A sub-cohort of included participants consumed a commercially available drink after an 8-h overnight fast. Each participant received the BOOST^®^ original drink (Nestle Health Science, Bridgewater, NJ, USA), providing 61.5 g of carbohydrates, 15 g of protein, and 6 g of fat. Blood samples were drawn using a venous catheter with stopcock apparatus at 0 (baseline), 15, 30, 45, 60, 75, and 90 min following the consumption of the drink [47].

### 2.5. Laboratory Variables

Fasting (at least 8 h of an overnight fast) and postprandial blood samples were used to measure several analytes. The MILLIPLEX^®^ MAP Human Metabolic Hormone Bead Panel—Metabolic Multiplex Assay, based on the Luminex xMAP (Luminex Corporation, Austin, TX, USA) technology, was used to measure leptin and ghrelin concentrations in plasma, following the assay protocol. The leptin/ghrelin ratio was calculated as leptin (ng/mL) × 1000/(ghrelin (pg/mL). Circulating hepcidin levels in plasma were measured using a solid phase enzyme-linked immunosorbent assay (DRG^®^ Hepcidin 25 Bioactive) EIA-5258, DRG International, Inc., Springfield, NJ, USA). Circulating ferritin levels were measured using an electrochemiluminescence immunoassay (©Roche Products and Roche Diagnostics Ltd., Auckland, New Zealand). Blood tests for HbA1c (mmol/mol) were conducted immediately after blood collection on fresh and never frozen blood using the boronate affinity chromatography assay (Trinity Biotech, Bray, Ireland), which was standardised to the Diabetes Control and Complications Trial reference assay and certified by the U.S. National Glycohaemoglobin Standardisation Program at the laboratory of Auckland City Hospital. FPG (mmol/L) tests were conducted in the same laboratory using an enzymatic colourimetric assay (F.Hoffmann-La Roche Ltd., Basel, Switzerland).

### 2.6. Other Variables

Anthropometric measurements, including height, weight, waist circumference, hip circumference, were collected in line with a standardised protocol [48]. Body mass index (BMI) was derived from weight and height as weight (kg)/(height (m) × height (m)). Basal metabolic rate was determined using the Harris-Benedict equation [49]. Recurrence of acute pancreatitis was dichotomised depending on whether participants had been re-admitted to hospital due to an acute pancreatitis attack (at least 30 days apart) from their first episode until the time of the ANDROMEDA project. The aetiology of acute pancreatitis was defined as biliary, alcohol-related, and other. Use of antidiabetic medications was ascertained from medical records and recorded as “yes” or “no”.

### 2.7. Statistical Analyses

Statistical analyses were performed using SAS 9.4. (SAS Institute Inc., Cary, NC, USA). Quantitative variables were expressed as mean and standard deviation (SD) or as median and interquartile range (IQR), as appropriate. Categorical variables were expressed as frequencies and percentages. The normality of variables was assessed using the hypothesis test (Shapiro–Wilk). The incremental area under the concentration-time curve (iAUC) for leptin and ghrelin were calculated using the trapezoidal method.

Multilinear regression models were used to analyse the associations between markers of iron metabolism (hepcidin and ferritin) and leptin, ghrelin, and the leptin/ghrelin ratio in the overall cohort (in both the fasted and postprandial states) and in the sub-groups stratified by iron intake and diabetes status. Markers of iron metabolism were analysed as the independent variables, whereas leptin, ghrelin, and the leptin/ghrelin ratio were the dependent variables. Linear regression models were built as follows: model 1—unadjusted; model 2—adjusted for age, sex, and body mass index; model 3—adjusted for age, sex, body mass index, and use of antidiabetic medications; model 4—adjusted for age, sex, body mass index, use of antidiabetic medications, aetiology of acute pancreatitis, and recurrence of acute pancreatitis. Significance tests were two-sided and *p* values were deemed statistically significant at the 5% level or lower.

## 3. Results

### 3.1. Characteristics of Study Participants

A total of 76 individuals in the fasted state and a sub-cohort of 34 participants in the postprandial state were analysed. The baseline characteristics of the sub-cohort were similar to the ones of the overall cohort, with the exception of age and fasting plasma leptin (Table 1). The median (IQR) of hepcidin and ferritin were 11.8 (8.5–18.9) ng/mL and 144.5 (94.5–257.5) ng/mL, respectively, in the overall cohort and 11.8 (8.6–18.1) ng/mL and 130.0 (83.0–199.0) ng/mL, respectively, in the sub-cohort. The mean ± SD of the iAUC of leptin and ghrelin were 27.62 ng/mL × min and 100.8 ± 162.2 pg/mL × min. Dietary iron intake was significantly higher in the “Sufficient” iron intake (48 (63%)) than the “insufficient” iron intake (28 (37%)) participants (*p* < 0.0001).

### 3.2. Associations of Markers of Iron Metabolism in the Overall Cohort

**Figure 1:**
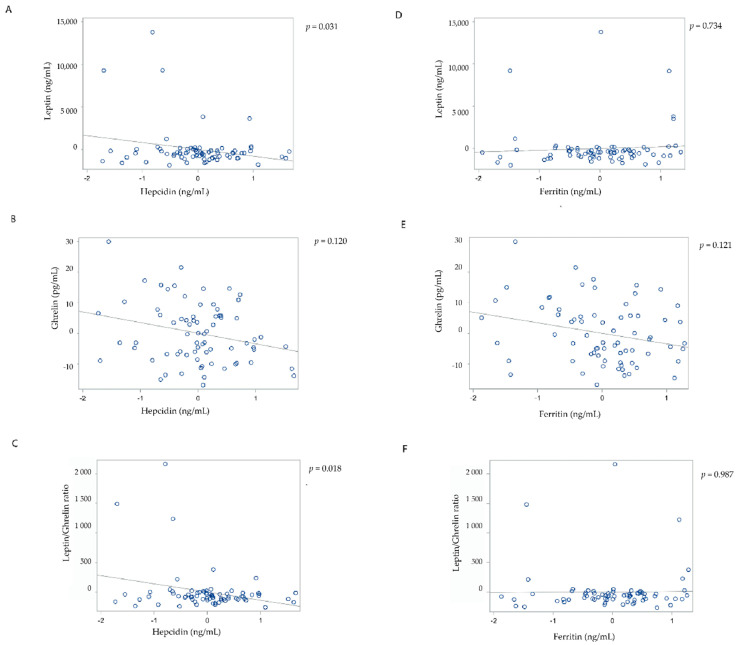
Associations between hepcidin and leptin (**A**), ghrelin (**B**), and the leptin/ghrelin ratio (**C**); and ferritin and leptin (**D**), ghrelin (**E**), and the leptin/ghrelin ratio (**F**) in the fasted state. All partial residual plots were adjusted for age, sex, body mass index, use of antidiabetic medications, aetiology of pancreatitis, and recurrence of pancreatitis. Hepcidin and ferritin data were transformed using natural logarithm transformation.

In the fasted state, hepcidin was significantly inversely associated with leptin in both the unadjusted and all the adjusted models. In the most adjusted model (model 4), the β coefficient ± SE was −883.45 ± 400.94; *p* = 0.031 (Table 2). Hepcidin was significantly inversely associated with the leptin/ghrelin ratio in both the unadjusted and all the adjusted models. In the most adjusted model (model 4), the β coefficient ± SE was −148.26 ± 61.20; *p* = 0.018. There was no significant association between hepcidin and ghrelin in all the models. Ferritin was not significantly associated with leptin, ghrelin, and the leptin/ghrelin ratio in both the unadjusted and adjusted models (Figure 1).

In the postprandial state, hepcidin was significantly directly associated with iAUC of ghrelin in the unadjusted model but not the adjusted models. In the unadjusted model (model 1), the β coefficient ± SE was 177.59 ± 81.60; *p* = 0.037. There was no significant association between ferritin and iAUC of ghrelin in all models. Further, iron metabolism markers were not significantly associated with iAUC of leptin, iAUC of ghrelin, and the iAUC leptin/iAUC ghrelin ratio (Table 2).

### 3.3. Associations of Markers of Iron Metabolism in the Sub-Groups Stratified by Dietary Iron Intake

In individuals with “sufficient” dietary iron intake, there were no significant associations between hepcidin and leptin, ghrelin, and the leptin/ghrelin ratio, in both the unadjusted and adjusted models (Table 3). The same was observed in individuals with “insufficient” dietary iron intake (Table 3). In individuals with “sufficient” dietary iron intake, ferritin was significantly inversely associated with ghrelin in the unadjusted model but not the adjusted models. In the unadjusted model, the β coefficient ± SE was −3.94 ± 1.88; *p* = 0.042 (Table 3). There were no significant associations between ferritin and leptin or the leptin/ghrelin ratio in both the unadjusted and adjusted models. In individuals with “insufficient” dietary iron intake, there were no significant associations between ferritin and leptin, ghrelin, and the leptin/ghrelin ratio, in both the unadjusted and adjusted models (Table 3).

### 3.4. Associations of Markers of Iron Metabolism in the Sub-Groups Stratified by Diabetes Status

In individuals with NODAP (n = 37), hepcidin was significantly inversely associated with leptin in the unadjusted and the most adjusted model. In the most adjusted model (model 4), the β coefficient ± SE was −806.09 ± 395.44; *p* = 0.050. Also, hepcidin was significantly inversely associated with the leptin/ghrelin ratio in both the unadjusted and adjusted models. In the most adjusted model (model 4), the β coefficient ± SE was −129.40 ± 59.14; *p* = 0.037 (Table 4). There was no significant association between hepcidin and ghrelin in both the unadjusted and adjusted models. There were no significant associations between ferritin and leptin, ghrelin, and the leptin/ghrelin ratio, in both the unadjusted and adjusted models (Table 4).

In individuals with T2DM (n = 23), hepcidin was significantly inversely associated with ghrelin in all but the most adjusted model. In model 3, the β coefficient ± SE was −6.24 ± 2.56; *p* = 0.028 (Table 4). There were no significant associations between hepcidin and either leptin or the leptin/ghrelin ratio, in both the unadjusted and adjusted models. There were no significant associations between ferritin and leptin, ghrelin, and the leptin/ghrelin ratio, in both the unadjusted and adjusted models (Table 4).

In individuals with normoglycaemia (n = 16), there were no significant associations between hepcidin and leptin, ghrelin, and the leptin/ghrelin ratio, in both the unadjusted and adjusted models. There were no significant associations between ferritin and leptin, ghrelin, and the leptin/ghrelin ratio, in both the unadjusted and adjusted models (Table 4).

## 4. Discussion

To the best of our knowledge, this is the first study in people with no diseases of iron metabolism reporting the associations between markers of iron metabolism and metabolic signals involved in the maintenance of energy balance (leptin, ghrelin, and the leptin/ghrelin ratio). Moreover, these associations were studied comprehensively by investigating both the fasted and postprandial levels of ghrelin and leptin. The present study also employed statistical models that took into account numerous possible confounding factors, including BMI, that accounted for the possible association between levels of leptin and the amount of adipose tissue [50]. The main finding of the present study was that hepcidin (but not ferritin) was significantly inversely associated with leptin and the leptin/ghrelin ratio in the fasted state (but not in the postprandial state) in both the adjusted and unadjusted models. This association remained significant in individuals with NODAP but not in those who had T2DM or normoglycaemia. The significance of the association with the leptin/ghrelin ratio was mainly driven by leptin and not by ghrelin (as evidenced by the lack of a significant association with ghrelin). Further, the stratification by habitual iron intake did not show any significant association between markers of iron metabolism and the leptin/ghrelin ratio, even after adjusting for demographics, anthropometrics, and other covariates.

The cellular regulation of leptin by iron has recently been suggested. The pioneering work by Gao et al., through studies in rodents and cultured cell models, demonstrated that cellular iron down-regulates transcription of leptin through phosphorylation of cAMP-responsive element-binding protein (i.e., CREB-activation) [26]. Specifically, treatment of 3T3-L1 adipocytes with iron led to CREB-activation and, when the iron-specific binding sites on the leptin promoter were mutated, the effect of iron status on leptin transcription was lost [26]. The iron-induced cellular changes in leptin triggered changes that led to rodent feeding behaviour where mice fed on high-iron chow had decreased leptin and increased food intake [26]. Although the precise mechanism of the link between iron and CREB-activation is unknown, iron has been shown to cause CREB-activation through decreasing levels of protein *O*-glycosylation (a nutrient-dependent mechanism of leptin regulation) [27]. Given that CREB is a key factor in the transition of fuel utilisation from feeding to fasting (leading to a gradual decrease of leptin) [51] and taking into account the evidence that hepcidin is involved in gluconeogenic sensing in the fasted state (by upregulation through PPARGC1A/CREBH potentiating reduced levels of circulating iron) [52], leptin could be a key mediator of the cross-talk between iron homeostasis and energy metabolism. Although we did not find a significant association between markers of iron metabolism and ghrelin, some cell culture studies showed that ghrelin increases the protein expression of ferroportin 1, internalisation of peripheral iron, and lowers ferritin levels [25]. The latter findings are yet to be reproduced in human studies.

Both iron and leptin have strong associations and are risk factors for derangements of glucose metabolism [53,54,55,56,57,58,59]. A key finding in the present study was the significant inverse association between hepcidin and leptin in individuals with NODAP, but not T2DM. Differing findings in the two types of glucose derangement states were not surprising given the evidence that both types are different. There is ample evidence that individuals with NODAP are at a higher risk of mortality [60], morbidities (including pancreatic cancer [61]), have poorer glycaemic control [62], differing gut hormone responses [47], and have different risks of mortality related to the use of antidiabetic medications [63,64] compared with those with T2DM. Further, NODAP is well characterised as derangements of glucose homeostasis after an attack of acute pancreatitis, whereas the underlying cause of T2DM is typically unknown [35]. In this study, the NODAP group included individuals who developed glucose derangements at an average of 20 months after hospitalisation, whereas T2DM included individuals with longstanding glucose derangements that began before hospitalisation for acute pancreatitis.

Leptin may be a candidate mediator for the link between iron and the development of NODAP because of its relationship with the factors unique to the pathogenesis of NODAP. First, there is evidence of a relationship between leptin and intra-pancreatic fat deposition [65]. One study showed a significant inverse association between leptin and intra-pancreatic fat deposition (determined with the use of magnetic resonance imaging), independent of abdominal fat distribution in individuals after an attack of acute pancreatitis [65]. Taking into account that individuals after an attack of pancreatitis have significantly higher intra-pancreatic fat deposition compared with healthy controls [66] and given the findings of significant inverse associations of intra-pancreatic fat deposition and indices of insulin sensitivity in non-obese NODAP individuals (but not in T2DM) [67], leptin could be a potential driver of glucose metabolism abnormalities in NODAP. Second, it is thought that leptin may share similar appetite regulating pathways with oxyntomodulin—a gut peptide that whose circulating levels are lower in individuals with NODAP [68]. Oxyntomodulin is derived from the posttranslational modification of proglucagon and appears to exert its anorectic actions, in part, through the glucagon-like peptide-1 receptor [69]. There is evidence suggesting that leptin interacts with the glucagon-like peptide-1 receptor in the vagal afferent neurons to induce satiety (by increasing calcium release) [70], potentially directly stimulating the action of oxyntomodulin. Considering that postprandial levels of oxyntomodulin were found to be markedly lower in NODAP compared with T2DM and healthy controls (independent of insulin secretion) [47], it is possible that leptin is an important factor in differentiating the pathogenesis of the two diabetes states. Individuals with NODAP have abnormal iron metabolism [71,72] and it is conceivable that the association between hepcidin and leptin observed in the present study could play a part in the complex pathophysiology of glucose derangements after acute pancreatitis.

Several limitations should be considered in interpreting the results of the present study. First, the cross-sectional nature of the study design precluded us from making a cause-effect inference. Longitudinal studies are warranted to investigate the relationship between iron metabolism and leptin with a view to establishing causality. Second, habitual iron intake was assessed using a food frequency questionnaire whose use might have led to measurement error. However, the EPIC Norfolk food frequency questionnaire is widely validated and gives a robust estimation of food and nutrient intake [43]. Third, undiagnosed diseases of iron metabolism that cause iron overload and deficiency might have affected the study’s associations. However, the median (IQR) of ferritin in our study population was 144.5 (94.5–257.5) ng/mL, negating iron overload and/or deficiency. Fourth, physiological variations between individuals undergoing the mixed meal test might have influenced postprandial responses of ghrelin and leptin. However, the variation of the basal metabolic rate of participants in the present study was minimal, as demonstrated by a mean ± SD of 1595.0 (307.0) kcal/day. Last, leptin and insulin play synergistic glucose-lowering roles and further regulate each other through the adipo–insular axis (where leptin diminishes insulin secretion by pancreatic beta cells and, in turn, insulin stimulates leptin secretion in the adipose tissue) [73,74]. Therefore, insulin levels might have affected the studied associations.

In conclusion, the present findings suggest the existence of an inverse association between hepcidin and leptin in humans with no overt disorders of iron metabolism. The leptin-iron interaction represents a largely unexplored pathway that may play a role in the pathogenesis of NODAP.

## Figures and Tables

**Table 1:** Characteristics of the study population.

Characteristic	Overall Cohort (n = 76)	Postprandial Sub-Cohort (n = 34)	*p*-Value
Age (years)	52.8 (14.9)	58.7 (6.4)	**0.011**
Men n (%)	51 (67)	24 (71)	0.850
Body mass index (kg/m^2^)	29.3 (6.3)	28.5 (6.4)	0.461
Waist circumference (cm)	99.6 (14.8)	99.2 (15.9)	0.973
Hip circumference (cm)	104.5 (14.9)	101.8 (13.3)	0.199
Waist-to-hip ratio	0.96 (0.08)	0.97 (0.07)	0.131
Fasting plasma leptin (ng/mL)	5.4 (2.5–16.2)	4.1 (2.4–13.7) ^a^	**0.010**
Fasting plasma ghrelin (pg/mL)	16.2 (8.2–25.5)	14.4 (9.0–27.7) ^a^	0.801
Dietary iron intake (mg/day)	9.4 (7.5–13.3)	10.4 (8.1–13.5)	0.592
Energy intake (kcal/day)	1628.0 (1226.5–2135.7)	1983.9 (1288.7–2382.3)	0.404
Alcohol (g/day)	1.6 (0–10.0)	3.3 (0–10.7)	0.531
Basal metabolic rate (kcal/day)	1626.9 (296.0)	1595.0 (307.0)	0.522
Fasting blood glucose (mmol/L)	5.5 (5.1–6.5)	5.5 (5.3–7.1)	0.215
Haemoglobin A1c (mmol/mol)	38 (35–41)	40 (37–44)	0.202
Recurrence of acute pancreatitis			0.760
No	55 (72)	23 (68)	
Yes	21 (28)	11 (32)	
Aetiology			0.441
Biliary	34 (41)	17(51)	
Alcohol-related	16 (20)	6 (18)	
Other	26 (39)	10 (30)	
Use of antidiabetic medications			0.563
No	66	30	
Yes	10	4	
Time since first pancreatitis attack (months)	19 (14–27)	19.6 (10–23)	0.060

*Footnotes*: Data are presented as mean ± standard deviation median, (interquartile range) or count. ^a^ At baseline (0 min) before the mixed meal test. *p*-values were from *t*-test (for continuous variables) and chi-square (for categorical variables) between the overall cohort and postprandial sub-cohort. *p*-values ≤ 0.05 are shown in bold.

**Table 2:** Associations between hepcidin, ferritin, and markers of energy balance in the fasted and postprandial states.

Marker	State	Model	Hepcidin			Ferritin		
			β	SE	*p*-Value	β	SE	*p*-Value
Leptin	Fasted	1	−839.02	343.39	**0.017**	−84.02	327.21	0.798
		2	−766.37	380.79	**0.048**	183.02	382.64	0.634
		3	−797.52	384.13	**0.042**	163.51	387.23	0.674
		4	−883.45	400.94	**0.031**	138.11	403.84	0.734
	Postprandial	1	39.79	53.48	0.463	84.05	33.39	0.444
		2	−11.84	64.91	0.857	97.40	43.02	0.247
		3	−13.59	66.44	0.840	98.49	44.14	0.171
		4	−12.21	71.67	0.866	83.95	45.71	0.420
Ghrelin	Fasted	1	−2.31	1.53	0.136	−2.19	1.42	0.126
		2	−2.53	1.69	0.139	−2.83	1.67	0.094
		3	−1.84	1.57	0.247	−2.07	1.56	0.189
		4	−2.47	1.57	0.120	−2.46	1.56	0.121
	Postprandial	1	177.59	81.60	**0.037**	28.02	72.70	0.703
		2	176.17	92.70	0.067	23.87	88.23	0.789
		3	178.64	98.09	0.079	15.01	92.35	0.872
		4	203.55	107.53	0.070	8.28	100.95	0.936
Leptin/ghrelin ratio	Fasted	1	−135.64	51.64	**0.011**	−25.01	50.84	0.624
		2	−131.09	57.03	**0.025**	4.20	59.49	0.944
		3	−136.32	57.65	**0.021**	0.62	60.48	0.992
		4	−148.26	61.20	**0.018**	−1.02	63.92	0.987
	Postprandial	1	508.93	3474.50	0.884	−658.64	1324.33	0.623
		2	−1703.07	4307.24	0.696	−1581.14	1818.22	0.394
		3	−1924.32	4549.60	0.676	−1587.83	1933.07	0.421
		4	−1501.89	4261.40	0.728	−939.35	1902.81	0.628

*Abbreviations:* β, regression coefficient estimate; SE, standard error of β. *Footnotes*: Postprandial data were incremental area under the concentration-time curve for leptin and ghrelin, calculated using the trapezoidal method. Hepcidin and ferritin were transformed using natural logarithm-transformation and fitted as the independent variables in linear regression models. Model 1: unadjusted; model 2: adjusted for age, sex, body mass index; model 3: adjusted for age, sex, body mass index, use of antidiabetic medications; model 4: adjusted for age, sex, body mass index, use of antidiabetic medications, aetiology of acute pancreatitis, recurrence of acute pancreatitis. *p*-values ≤ 0.05 are shown in bold.

**Table 3:** Associations between hepcidin, ferritin, and markers of energy balance in the fasted state, stratified by dietary intake.

Marker	Iron Intake	Model	Hepcidin			Ferritin		
			β	SE	*p*-Value	β	SE	*p*-Value
Leptin	“Insufficient” iron intake	1	−560.17	527.67	0.299	384.06	457.41	0.409
		2	−299.91	693.95	0.670	995.04	571.63	0.096
		3	−289.77	708.71	0.690	998.47	583.61	0.103
		4	−456.75	718.98	0.533	975.97	634.17	0.141
	“Sufficient” iron intake	1	−678.34	460.41	0.148	86.43	400.90	0.830
		2	−673.04	454.84	0.147	−3.74	456.76	0.994
		3	−714.47	469.01	0.136	−22.07	476.69	0.963
		4	−496.29	529.24	0.354	53.15	482.07	0.913
Ghrelin	“Insufficient” iron intake	1	−0.56	2.72	0.838	−0.66	2.44	0.788
		2	−1.68	3.48	0.635	−1.81	3.23	0.582
		3	−1.68	3.52	0.640	−1.64	3.30	0.625
		4	−2.89	3.37	0.403	−2.99	3.16	0.358
	“Sufficient” iron intake	1	−4.11	2.22	0.070	−3.94	1.88	**0.042**
		2	−3.98	2.30	0.091	−4.14	2.26	0.075
		3	−2.33	1.95	0.239	−2.21	1.95	0.263
		4	−3.20	2.17	0.148	−2.06	1.99	0.308
Leptin/ghrelin ratio	“Insufficient” iron intake	1	−76.15	71.96	0.301	60.77	65.09	0.360
		2	−50.91	93.83	0.593	139.10	82.51	0.107
		3	−50.89	95.83	0.601	137.54	84.90	0.121
		4	−74.00	97.20	0.456	119.70	88.82	0.195
	“Sufficient” iron intake	1	−107.56	724.54	0.145	7.78	64.33	0.904
		2	−105.27	71.71	0.150	−6.99	73.44	0.925
		3	−111.16	73.89	0.141	−9.88	76.52	0.898
		4	−696.89	85.15	0.418	10.67	77.88	0.892

*Abbreviations:* β, regression coefficient estimate; SE, standard error of β. *Footnotes*: Hepcidin and ferritin data were transformed using natural logarithm transformation and fitted as the independent variables in linear regression models. Model 1: unadjusted; model 2: adjusted for age, sex, body mass index; model 3: adjusted for age, sex, body mass index, use of antidiabetic medications; model 4: adjusted for age, sex, body mass index, use of antidiabetic medications, aetiology of acute pancreatitis, recurrence of acute pancreatitis. Insufficient and sufficient iron intake was defined in accordance with the recommendations of the Institute of Medicine [44]. *p*-values ≤ 0.05 are shown in bold.

**Table 4:** Associations between hepcidin, ferritin, and markers of energy balance in the fasted state, stratified by diabetes status.

Marker	Model	Diabetes Status	Hepcidin			Ferritin		
			β	SE	*p*-Value	β	SE	*p*-Value
Leptin	1	Normoglycaemia	−1964.10	1549.34	0.226	927.80	1697.09	0.595
	2		−2813.72	2088.47	0.205	1484.94	2131.15	0.504
	3		−2809.74	2187.83	0.228	1464.94	2332.29	0.547
	4		−3051.06	2258.88	0.210	1550.02	2407.59	0.540
	1	T2DM	185.92	283.34	0.519	108.88	283.22	0.705
	2		400.71	265.85	0.149	362.17	272.18	0.201
	3		351.80	275.21	0.218	332.78	275.19	0.244
	4		126.98	366.45	0.734	191.25	317.29	0.556
	1	NODAP	−818.31	346.00	**0.024**	−336.33	301.00	0.272
	2		−752.19	389.38	0.062	−214.77	412.01	0.606
	3		−754.03	395.13	0.066	−212.73	418.32	0.615
	4		−806.09	395.44	**0.050**	−302.96	415.91	0.472
Ghrelin	1	Normoglycaemia	−1.69	3.86	0.669	−3.59	3.65	0.345
	2		3.55	5.04	0.496	−5.18	4.50	0.280
	3		3.58	5.23	0.510	−6.08	4.76	0.237
	4		3.78	5.08	0.476	−5.53	4.73	0.281
	1	T2DM	−7.50	2.78	**0.014**	−5.94	3.47	0.104
	2		−7.82	2.96	**0.018**	−6.40	3.77	0.110
	3		−6.24	2.56	**0.028**	−5.24	3.17	0.121
	4		−7.02	3.70	0.080	−4.41	4.17	0.311
	1	NODAP	0.03	1.93	0.986	−1.02	1.58	0.525
	2		−0.39	2.12	0.856	−2.12	2.10	0.321
	3		0.61	3.12	1.856	−2.10	2.13	0.332
	4		−1.27	2.00	0.531	−2.19	1.96	0.274
Leptin/ghrelin ratio	1	Normoglycaemia	−295.69	232.25	0.224	118.84	255.85	0.651
	2		−439.54	305.44	0.178	190.01	317.45	0.564
	3		−439.25	320.27	0.200	191.90	347.43	0.596
	4		−478.67	328.58	0.179	202.71	357.65	0.589
	1	T2DM	15.98	26.43	0.553	71.70	30.43	0.816
	2		29.69	25.74	0.266	25.44	30.39	0.416
	3		23.34	26.34	0.389	20.86	30.37	0.503
	4		−8.92	35.63	0.806	−8.41	37.23	0.825
	1	NODAP	−133.41	51.65	**0.014**	−63.75	45.06	0.166
	2		−121.13	58.02	**0.045**	−49.16	61.58	0.431
	3		−121.44	58.85	**0.048**	−48.81	62.51	0.441
	4		−129.40	59.14	**0.037**	−62.19	62.30	0.326

*Abbreviations:* β, regression coefficient estimate; SE, standard error of β; T2DM, type 2 prediabetes/diabetes mellitus; NODAP, new-onset prediabetes/diabetes after acute pancreatitis. *Footnotes*: Hepcidin and ferritin data were transformed using natural logarithm transformation and fitted as the independent variables in linear regression models. Model 1: unadjusted; model 2: adjusted for age, sex, body mass index; model 3: adjusted for age, sex, body mass index, use of antidiabetic medications, aetiology of acute pancreatitis, recurrence of acute pancreatitis. *p*-values ≤ 0.05 are shown in bold.

## Data Availability

All data analysed are included in the present manuscript.
